# Survival with primary lung cancer in Northern Ireland: 1991–1992

**DOI:** 10.1007/s11845-023-03465-9

**Published:** 2023-08-22

**Authors:** Gilbert MacKenzie

**Affiliations:** Formerly of the Centre of Biostatistics, University of Limerick, Limerick, Ireland

**Keywords:** Lung cancer, Mortality, Survival, Treatment, Statistical modelling, Screening table

## Abstract

**Abstract:**

Lung cancer is a major cause of death in Western countries, but survival had never been studied in Northern Ireland (NI) on a population basis prior to this study.

**Aims:**

The primary aims were to describe the survival of patients with primary lung cancer, evaluate the effect of treatment, identify patient characteristics influencing survival and treatment and describe current trends in survival.

**Methods:**

A population-based study identified all incident cases of primary lung cancer in NI during 1991–2 and followed them for 21 months. Their clinical notes were traced and relevant details abstracted. Survival status was monitored via the Registrar General’s Office, and ascertainment is thought to be near-complete. Appropriate statistical methods were used to analyse the survival data.

**Results:**

Some 855 incident cases were studied. Their 1-year survival was 24.5% with a median survival time of 4.7 months. Surgical patients had the best 1-year survival, 76.8%; however, adjustment suggested that about half of the benefit could be attributed to case-mix factors. Factors influencing treatment allocation were also identified, and a screening test showed the discordance between ‘model’ and ‘medic’: 210 patients were *misclassified*. Finally, the current trend in 1-year survival observed in the Republic of Ireland was best in the British Isles.

**Conclusions:**

Overall, survival remains poor. The better survival of surgical patients is due, in part, to their superior case-mix profiles. Survival with other therapies is less good suggesting that the criteria for treatment might be relaxed with advantage using a treatment model to aid decision-making.

## Introduction

In Northern Ireland (NI), there was a three-fold increase in numbers of deaths from lung cancer in males and a ten-fold increase in female deaths during the period 1950 to 1990 [1]. However, historically, the Epidemiology of lung cancer in NI has been poorly described, and research reports are relatively sparse.

Dean [2] found that NI had a lower mortality rate from lung cancer compared to other areas in Great Britain. Patterson and Kee [3] in their study of trends in mortality during 1979–1988 reported a 3% annual increase in the rate amongst females (the corresponding rate for males was virtually unchanged). Wilkinson [4], measuring population incidence of the condition for the first time, showed that NI had the lowest rates of any region of the British Isles.

At the time of the original study, there was a similar dearth of information on patient survival rates in NI. An extensive literature search revealed only one paper: Smiley and Cheeseman [5] considered the survival times of lung cancer patients referred for surgery. Significantly better survival was found amongst patients with squamous carcinoma, and the 1-year survival after surgery was 49%. Accordingly, it was decided to study survival amongst incident cases of lung cancer occurring in the population of NI over a 1 year period. This project was designated the NI Lung Cancer Study (NILCS).

In 1991–2, specific lung cancer treatment was centralised in NI: radiotherapy and chemotherapy in Belvoir Park Hospital, Belfast, and surgery at the Royal Victoria Hospital, Belfast. Chemotherapy for small cell carcinoma of the lung was also administered by respiratory physicians. Multidisciplinary lung cancer meetings were being held in Belfast from the 1970 s onwards. Not every hospital had a specialist respiratory physician, and referrals from these units were made to a respiratory specialist or directly to the surgical team in Belfast or to clinical oncology either at Belvoir Park Hospital or at one of their peripheral clinics. In the West of the province, there was a specialist respiratory physician based at Altnagelvin Hospital. Cases from Tyrone County Hospital and the Erne Hospital were also frequently referred to Belfast. Some patients, considered too ill to undergo surgery, chemotherapy and/or radiotherapy, were often not referred.

National guidelines were not available in 1991–2, and practice in NI was based mainly on national consensus, on the literature and on local experience. The British Thoracic Society was not to publish guidelines on the selection of patients for lung cancer surgery for another decade [6]. Inevitably, there was variability in medical practice. However, the situation in NI was similar to that prevailing elsewhere in the UK at the time.

## Aims

The primary aim of this paper is to describe survival amongst incident cases of lung cancer in NI in 1991–2 and to identify (if possible) factors which are predictive of good survival with a view to evaluating the effect of treatment. Because the decision to treat is based on the same set of risk factors included in the survival model, this goal requires us to investigate the performance of the *treatment model* which yields the probability that a patient receives therapy. Thus, the effect of factors included in the *survival model* may have been modified (for example, reduced) by this prior selection process (note the chronology: *selection* $\rightarrow$ *treatment* $\rightarrow$ *survival* ). Accordingly, we studied,* inter alia,* the interplay between these two models. And finally, we aimed to describe current trends in 1-year survival from primary lung cancer in the 5 countries of the British Isles.

## Methods

### Population studied

All persons resident in NI who were newly diagnosed as having primary lung cancer (ICD 162 9th. Revision) between 1 October 1991 and 30 September 1992 (*incident cases*) were studied [7]. Thus framed, the definition specifically excludes cases of mesothelioma (ICD163) or secondary lung cancer.

### Outcomes

The primary outcome is survival time, defined as ‘time from diagnosis to death from any cause’, i.e. ‘all cause’ mortality. In the NILCS data, we found that ‘all cause’ was almost identical to ‘cause specific’ mortality. Accordingly, hereafter, we dispense with the need to consider ‘cause specific’ mortality. Follow-up status was determined using death certificates (Registrar General NI) and/or contact with the patient’s general practitioner.

The secondary outcome was the estimated probability of each patient receiving curative therapy in the study. This probability is derived from the treatment model constructed using the same pool of factors contending for inclusion in the survival model, thus enabling one to investigate, *inter alia*, the consistency between the two models.

### Ascertainment

Incident cases were identified using a comprehensive multi-source notification scheme described in detail elsewhere [4, 7]. When the study was undertaken, Cancer Registration had not been implemented in NI. The multi-source notification scheme used in the study covered general practitioners, hospital physicians and surgeons, radiotherapists, pathology laboratories, the office of the Registrar General (NI) and the NI Cancer Notification scheme. This latter scheme was in operation prior to the introduction of cancer registration in NI. When it was evaluated in 1991/92, it was found to have identified only 40.1% of incident cases of lung cancer [4].

### Classification of lung cancer

Due to the diversity of investigations undertaken, the incompleteness of hospital charts and the absence of a functioning Cancer Registry routinely recording the standard data-set [8], it was impossible to employ any of the internationally accepted staging rubrics successfully. Accordingly, the extent of the disease has been classified on the basis of the available information in the patients’ clinical notes.

### Follow-up period

Cases were followed until the study terminated on May 30, 1993. Thus, the maximum possible follow-up time was 20 months for a person entering the study on October 1, 1991, and remaining ‘on-study’ until the termination date. Those who were still alive on May 30, 1993, were ‘censored’, i.e. their potential time to death was greater than their time on study.

### Risk factors

Patient details were obtained, and charts were reviewed at the hospitals concerned and recorded data on a specially designed two-part questionnaire. The following factors were selected for study in order to determine their influence, if any, upon survival: (a) patient characteristics—age, sex, marital status, social class, county of residence; (b) disease status/markers—cell type, WHO performance status [9], extent of metastases (if any), alkaline phosphate, sodium, albumen levels; and (c) treatment details. Further details of the categorisation of the various factors studied are shown in the accompanying tables. A subset of nine risk factors was included in the survival analysis described later.

### Treatment

Amongst the several factors recorded, treatment was considered to be the most important and was categorised as surgery, radiotherapy, chemotherapy, combined radiotherapy and chemotherapy or palliative treatment. Treatment was further classified into (a) potentially curative treatments, namely surgery, radical radiotherapy and chemotherapy (± radiotherapy) and (b) palliative or supportive treatments. The latter group encompasses patients who received palliative radiotherapy, where the object of treatment is purely to relieve symptoms, and patients who received no active therapeutic intervention.

Treatment has a different status from the other factors as it is a medical intervention involving a multi-criteria selection process based, in part, on the other measured factors (e.g. age, WHO performance status).

### Statistical methods

The analysis was conducted in several stages. Overall survival curves [10] were computed for categorical risk factors singly. The log rank test [11] was used to test the homogeneity of survival curves within subclasses of these factors.

We intended to use Cox’s proportional hazards (PH) regression model [12] to identify the independent effect of factors studied simultaneously. However, not all of the survival data followed the PH assumption. In particular, survival in the treatment categories was not PH. The non-PH Weibull multi-parameter regression (MPR) survival model [13] was used instead. The hazard function for the Weibull distribution is

1 $$\begin{aligned} \lambda (t) = \lambda \gamma t^{\gamma -1} \end{aligned}$$

where $\lambda >0$ is the *scale* parameter and $\gamma >0$ is the *shape* parameter. The MPR specification is then $\lambda = \exp (x'\beta ) \, \textrm{and} \, \gamma = \exp (x'\alpha )$ i.e. one predictor for the scale and another for the shape, unlike Cox model, which supports only $x'\beta$. The model was fitted by Maximum Likelihood in the R Core Team [14] software package mpr [15]. The Weibull MPR model defaults to a standard Weibull (PH) model when the there are no covariates in the shape parameter.

A multiple linear logistic model [16] was used to identify factors predictive of a patient receiving therapy in which the probability of being treated ($Y=1$) is given by

2 $$\begin{aligned} \textrm{Pr}(Y=1) = \exp (x'\beta ) / [1+\exp (x'\beta )] \end{aligned}$$

where $x' =(x_1, \dots , x_p)$ is a row vector of covariates for a patient and $\beta$ is a column vector of *p* parameters measuring the influence of the covariates on the probability of treatment. The parameters were estimated by the method of ML using the binary logistic regression procedure in SPSS (Version 26) [17]. Using the estimated prediction model, a screening table was constructed, from which the number of false positives (patients predicted to receive active treatment, but who did not) was found. Survival in this group was compared to survival in patients who were treated in accordance with model predictions (true positives).

The 5% level of statistical significance, which should be regarded merely as a nominal reporting level, was used in performing statistical tests and constructing confidence intervals (CIs).

## Results

### Data analysed

The study identified a total of 900 incident cases of primary lung cancer in NI during 1991–2. Of these, 693 (77.0%) were deaths. The registered cause of death was ‘lung cancer related’ in 646 (93.2%), ‘unrelated’ in 44 (6.4%) and unknown in 3 (0.4%). Despite extensive enquiries, the outcome could not be determined in 25 (of the 900) incident cases, and a further 20 incident cases were diagnosed at post-mortem. Accordingly, these latter 45 (5.0%) were excluded from the survival analysis which was then based on 855 cases. Of these, 673 (78.7%) patients had died by the censoring date (30th May, 1993), leaving 182 (21.3%) censored.

**Fig. 1:**
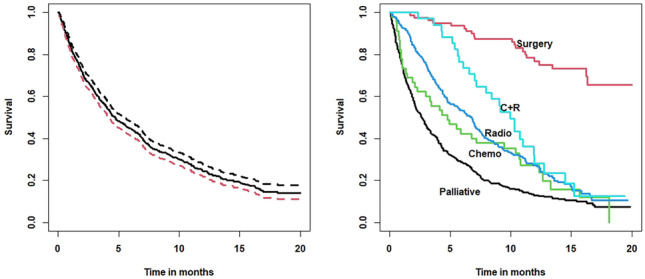
NI Lung Cancer Study: Kaplan Meier survival curves showing (a) overall survival with 95% CI (left panel) and (b) survival by treatment (right panel)

### Survival by treatment

Figure 1 (left panel) shows the overall Kaplan Meier (KM) survival curve for the study data over the follow-up period. Half the patients were dead by 4.7 months, and the survival at 1-year was 24.5%.

The right panel of Fig. 1 shows the KM estimator by treatment category (surgery, chemotherapy, radiotherapy, chemo. + radio., palliative care). The 1-year survival percentages were as follows: (a) surgical patients (79/855) had the best survival, 76.8% (95% CI = 67.6%$-$87.2.%), (b) chemotherapy patients (45/855) had a survival of 23.7% (95% CI = 13.5%$-$41.7%), (c) radiotherapy patients (256/855) had a survival of 26.5% (95% CI = 21.4%$-$32.9%), (d) combined therapy (patients 34/855) had a survival of 28.1% (95% CI = 15.7%$-$50.2%), and finally, (e) palliative care patients (441/855) had the worst survival, 13% (95% CI = 10.1%$-$16.8%).

Of the 256 patients receiving radiotherapy, only 21 (8.2%) were treated with curative intent, and their median survival was 13.6 months. Thus, the remaining 235 patients were treated palliatively in order to relieve symptoms (median = 6.2 months). When amalgamated with the 441 palliative patients, the total number receiving palliative care was 676 (79.1%) of the 855 incident cases studied, a finding due, principally, to late diagnosis. In the main analyses, all patients receiving radiotherapy have been treated as one group.

Only 34 patients received combined treatment, but their survival over the first year was superior to that of patients receiving chemotherapy or radiotherapy alone (but note the width of the confidence interval with *n* = 34). However, survival declined sharply after 6 months in the combined therapy group. Radiotherapy patients had slightly better survival than chemotherapy patients for the first 8 months, when the survival curves crossed, and were indistinguishable thereafter (Fig. 1). The difference in 1-year survival between surgical and non-surgical patients is striking.

#### Modelling survival

**Fig. 2:**
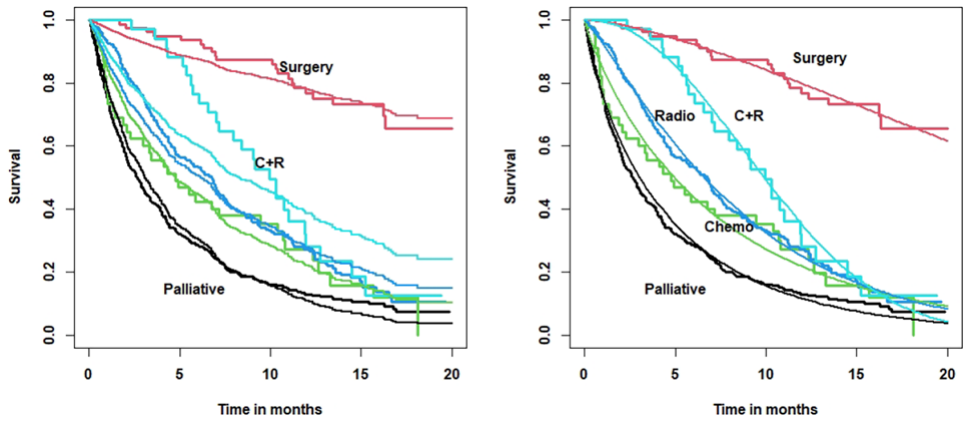
NI Lung Cancer Survival Study: Kaplan Meier curves showing (a) Weibull PH predicted tracks (left panel) and (b) non-PH Weibull MPR predicted tracks (right panel) by treatment category

From Fig. 2 (left panel), it is clear that these data do not follow the PH assumption: note the lack of fit in the combined therapy group and the late crossings of the chemotherapy and radiotherapy survival curves. However, the right panel shows that the non-PH Weibull MPR model fits the data well as evidenced by the excellent fit to the combined therapy track. We compared the Weibull PH model to the non-PH Weibull MPR model, and the AIC for the non-PH model was 43 units lower, indicating a significantly superior fit (the lower the better). No model is perfect, but the evidence supports the use of the non-PH Weibull MPR model.

Table 1 shows the Maximum Likelihood estimators for the treatment coefficients in the hazard function and their standard errors for the fitted Weibull MPR model. Everything is measured relative to palliative care which has coefficients of zero in the scale and shape components (omitted from Table 1). The coefficients in the scale component are all negative showing that treatment reduces the hazard and improves survival.

**Table 1:** NILCS Maximum Likelihood estimators and standard errors (s.e.) for the Weibull MPR model including treatment

Treatment	Scale			Shape	
	mle ($\beta$s)	s.e.		mle($\alpha$s)	s.e.
Intercept	-1.280	0.080		-0.194	0.041
Surgery	-3.909	0.833		0.593	0.205
Chemotherapy	-0.499	0.319		0.072	0.143
Radiotherapy	-1.261	0.188		0.337	0.072
Combined (C+R)	-4.058	0.883		0.968	0.164

NB: Reference category = palliative care with both coefficients = 0

With the exception of chemotherapy, of all the effects in the scale reach statistical significance: surgery and combined therapy being particularly efficacious. In the shape component, the treatment coefficients are all positive, thereby increasing the hazard and indicating that the beneficial effects of treatment wane with time. In particular, in the shape component, the combined therapy effect is relatively large, indicating a more rapid decline in survival.

##### Remark

Recall that these data are observational, and consequently, the comparisons between treatment categories are not protected by randomisation. The analysis of treatment alone is thus problematical as the magnitude of any benefit observed is unlikely to be wholly attributable to treatment.

### Factors influencing survival

Next, we investigated which of the following nine covariates: (1) treatment, (2) age, (3) sex, (4) WHO status, (5) celltype, (6) sodium level, (7) albumen level, (8) metastases and (9) smoking status, measured at the time of diagnosis were predictive of survival.

####  Overview

Table 2 shows the result of fitting the stepwise forward Weibull MPR model to the data. Several interesting features emerge. First, we note that the algorithm selects 6 variables in the scale component and 4 variables in the shape component. Three of the variables—treatment, metastases and albumen level—influence both the scale and shape. WHO status, cell type and sodium level influence the scale, but not the shape, and smoking influences the shape, but not the scale. The fact that 4 variables appear in the shape component means that these variables do *not* satisfy the PH assumption. We note that the variables selected do not include age nor sex, and one is led to the conclusion that these variables do not influence survival, *given* the presence of the others in the model (but see a fuller interpretation below).

#### Multi-factor interpretation

Since all of the factors in the model are on the same scale, the magnitude of the coefficients is a good guide to the importance of the effects. Looking at the scale components first, we see that treatment reduces the hazard (negative coefficients). While the beneficial effects of combined therapy, surgery and radiotherapy are large and each reaches statistical significance, that of chemotherapy does not. These beneficial effects wane with time due to the positive shape coefficients. For example, in the scale, the effect of surgery is mle = $-$1.829, s.e. = 0.757, $z=2.41, p= 0.016$, which is offset in the shape by mle = +0.191, s.e.= 0.195, $z = 0.98, p > 0.05$. In this case, the offset is not statistically significant from zero, so we expect only a slight increase in the hazard function with time amongst patients receiving surgery. Contrast this finding with that of combined therapy where the large beneficial effect in the scale (mle = $-$4.08) component is offset by +0.837 (*z* = 5.30, $p \ll 0.05$) in the shape, leading to an increasing hazard with time.

**Table 2:** NILCS survival model - Maximum Likelihood estimators and standard errors (s.e.) for the Weibull MPR model - stepwise forward solution pertaining to 9 covariates including treatment

Covariates	Scale			Shape	
	mle ($\beta$s)	s.e.		mle ($\alpha$s)	s.e.
Intercept	-4.029	0.351		0.094	0.101
*Treatment*					
Surgery	-1.829	0.757		0.191	0.195
Chemotherapy	-0.341	0.328		-0.036	0.129
Radiotherapy	-0.894	0.193		0.254	0.069
Combined (C+R)	-4.088	0.914		0.837	0.158
*Metastases*					
Present	1.465	0.278		-0.234	0.076
Unknown	0.936	0.298		-0.182	0.088
*Albumen level*					
< 3.5 g/l	0.707	0.151		-0.116	0.058
Unknown	0.327	0.261		0.054	0.089
*WHO status*					
Light work	0.130	0.185		-	-
Unable to work	0.614	0.187		-	-
>50% walking	1.077	0.200		-	-
Bed/chair bound	1.624	0.288		-	-
*Cell type*					
Small cell	0.707	0.157		-	-
Adeno. ca.	0.334	0.140		-	-
Other	0.235	0.103		-	-
*Sodium*					
< 136mmol/l	0.324	0.085		-	-
Unknown	-0.051	0.219		-	-
*Smoking*					
Current	-	-		0.171	0.053
Ex	-	-		0.151	0.055
Unknown	-	-		0.089	0.124

NB: AIC = 3679.65; - = factor not present in scale or shape. Coefficients (i.e. mles): +signs increase the hazard, and -signs decrease it, mle significant if $\mid z\mid$ = $\mid mle/s.e.\mid \,\, \ge$ 2

**Fig. 3:**
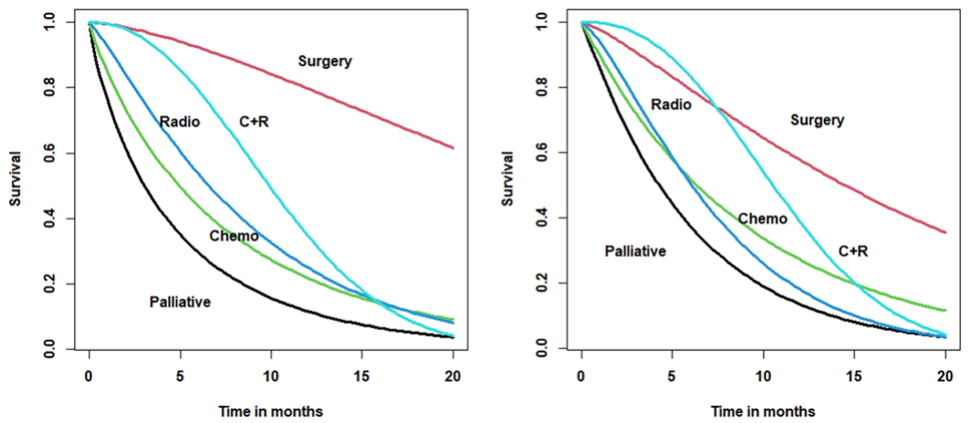
NI Lung Cancer Study: non-PH Weibull MPR survival model showing (a) unadjusted predicted survival tracks (left panel) and (b) adjusted survival tracks (right panel) by treatment categories

### The effect of adjustment

The multi-factor findings are important because they allow one to compare the crude treatment effect in Table 1 with the adjusted effect in Table 2. Comparing the scale coefficients for treatment in Tables 1 and 2, the effect of adjustment is to reduce the benefit attributable to surgery, chemotherapy and radiotherapy, but *not* to combined therapy. This latter finding suggests that the observed combined therapy effect holds irrespective of case-mix. In contrast, approximately half the benefit attributed to surgery (Table 1) can be explained by the non-treatment factors in the model (Table 2). Finally, we note that chemotherapy does not reach statistical significance in any component of either model.

The effects of the adjustment for modelled factors are best seen graphically. Figure 3 shows the estimated unadjusted treatment survival tracks (left panel) and the adjusted treatment tracks (right panel). The adjusted tracks are for an individual following a given treatment track who is *average* with respect to the other covariates. The reduction in benefit attributable to surgery at 1-year is evident in the right panel. Adjustment for any combination of factors in the model (Table 2) is possible and will repay study (e.g. predicting survival in an older surgical patient).

## Treatment model

We turn now to investigate which patients received treatment. Of particular interest is the consistency of decisions made and how the treatment model affects the interpretation of the survival model shown in Table 2.

**Table 3:** NILCS treatment model -multi-factor logistic model, variables selected by stepwise forward analysis

Variable (*n*)	mle	s.e.	*p*
Intercept	-3.0315	0.5786	<0.001
1. WHO			
*Normal* (78)	-	-	-
Light work (278)	-0.3051	0.3371	0.3654
No work (286)	-0.9918	0.3369	0.0032
W/B/C^a^ (213)	-2.2077	0.3749	< 0.001
2. Age-group			
*< 40* (32)	-	-	-
40- (89)	0.0888	0.5497	0.8716
50- (311)	-0.7097	0.4914	0.1487
60- (299)	-1.2798	0.4958	0.0098
70- (124)	-1.5085	0.5497	0.0061
3. Cell			
*Squamous* (247)	-	-	-
Small (121)	0.2771	0.2745	0.3127
Adeno ca (108)	-0.7248	0.2767	0.0088
Other (379)	-0.9961	0.2088	< 0.001
4. Metastases			
*No Met* (188)	-	-	-
Met (428)	-0.3840	0.2229	0.0849
Missing (239)	-1.1209	0.2618	< 0.001
5. Albumen g/l			
$\ge$ *35* (458)	-	-	-
< *35* (315)	-0.4989	0.1851	0.0070
Missing (82)	-0.9589	0.3360	0.0043

The reference categories are in italics

^a^ = more than 50% waking time bed or chair bound

### Selection for treatment

Prior to performing the survival analysis, the covariates were included in a multiple linear logistic model of the probability of receiving treatment given by Eq. (2). Table 3 shows the 5 factors selected into the logistic treatment model. The probability of receiving therapy declines (negative coefficients) with worsening WHO status, increasing age, the presence of non-small cell cancer, metastatic disease and low levels of albumen.

Thus, the treatment model differs from the survival model in a number of ways. First, notice the age-dependence in the treatment model and its absence in the survival model. This finding suggests that any potential age-effect in the survival model (Fig. 5, panel (b)) is being subsumed within the treatment variable. We checked this conjecture in the survival model by fitting age first and then the treatment variable. The significant effects observed in the age model disappeared completely when the treatment variable was added, thereby (indirectly) supporting the conjecture. This, however, is not true for WHO status, cell type, metastases and albumen, factors which influence treatment, but which also exercise an independent effect on survival, indicating that their effects are not (wholly) accounted for by the treatment variable. Finally, we note that the treatment model does not depend on the sodium nor smoking measures which appear in the survival model.

Given that treatment allocation is a multi-criteria decision, one useful way of interpreting the effects of *non-treatment* factors appearing in the survival model is that their influence cannot be explained solely by the inclusion of treatment. Clearly, both the survival model and the treatment model are required for a correct interpretation of the data, since, without the treatment model, the survival findings might lead one to conclude that there is no age effect at all.

**Fig. 4:**
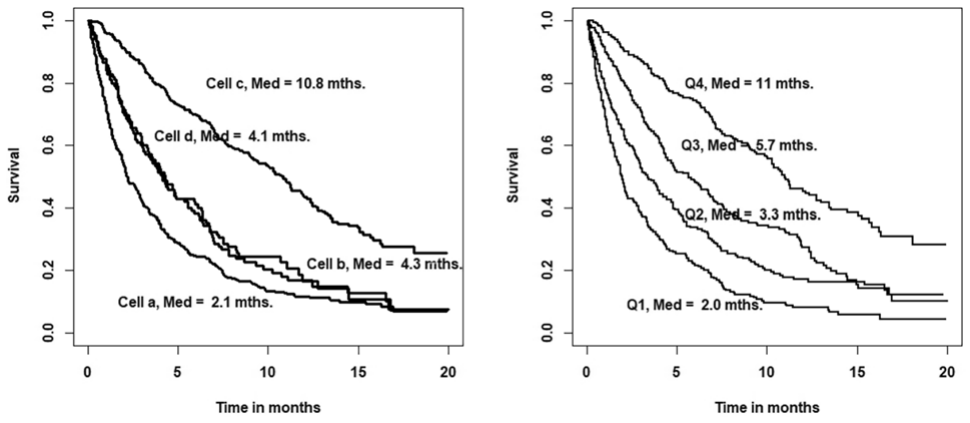
NI Lung Cancer Study: showing (a) KM unadjusted predicted survival tracks (left panel) for each cell of the screening table and (b) KM survival tracks by quartile of estimated probability of treatment (right panel)

### Elderly patients

There is an extensive body of literature indicating that older lung cancer patients are under-treated and hence have higher mortality and poorer survival [18–22].

Table 3 provides *prima facie* evidence of ‘ageism’, i.e. of younger patients being selected (significantly) more frequently for treatment as part of routine clinical practice, when allowing for other selection factors. We have already seen how* advantageous *selection is and the point at issue is whether the criteria can be relaxed to treat and hence improve survival in more (perhaps older) patients.

Because of this selection, part, or all, of the influence of age is embodied in the treatment variable, and Table 2 and the analysis above show that, in these data, there is consequently no additional age effect in the survival model.

**Table 4:** NILCS screening table - based on the estimated probabilities of treatment

	Model prediction		
Truth	Do not treat	Do treat	Total
Not treated	**a** 336	**b** 105	441
Treated	**d** 105	**c ** 309	414
Total	441	414	855

Cut-off prob. = 0.5

Sensitivity = c/(c+d) = 74.6%

Specificity = a/(a+b) = 76.2%

### Screening table and survival

First, we used the model to estimate the probability of each patient receiving therapy and constructed an epidemiological screening table based on these probabilities. The results are in Table 4, which shows the sensitivity (75%) and specificity (76%) rates. The false positive rate, 23.8%, and the false negative rate, 25.4%, are rather high. The symmetry of the table is also interesting, since, given that an error will be made, a false positive is as likely as a false negative in these data (i.e. the probability of each is $\approx 1/2$).

One interpretation of Fig. 4 is that the survival of the 105 patients who were treated ‘against’ the model’s prediction (false negatives, cell **d**) was improved: the median rose from 2.1 to 4.3. Consequently, it is natural to conjecture that the survival of patients who were not treated against the model’s prediction (false positives, cell **b**) might also be improved. Accordingly, any review of clinical decisions reached should be focussed on this group.

Some attention needs to be paid to the numbers involved. Only 79 highly selected patients received surgery, and their survival is very good relative to other treatment groups (Fig. 2, right panel). However, there are 309 patients in Table 3 who were treated in accordance with the model’s prediction (cell **c**). This larger group included 75 of the 79 patients who received surgical treatment as well as 38 of the 45 who received chemotherapy, 164 (64.1%) of the 256 who received radiotherapy and 32 of the 34 who received combined therapy.

Clearly, the model identifies, correctly, a group of patients destined to have improved survival on treatment, reinforcing the view that the false positive group (cell **b**) is the target for review (especially those in the radiotherapy group).

Finally, Fig. 4 shows how the derived treatment model graduates survival well, noting (a) the separation between true positives and true negatives (in left panel) and (b) by quartile of estimated risk (in right panel). Recall, that there are 214 patients on the upper survival track in the right hand panel (including the majority of surgical patients).

## Trends in survival

Much has changed since 1991–2, but unfortunately, lung cancer survival has shown only modest improvement in the UK. It will be recalled that the NILCS estimated the 1-year survival to be 24.5%. In 2010, the author was commissioned to assess the methodology on which the NICE guideline on lung cancer for the UK was based [23]. NICE’s observed survival curve, based on selected English data, was similar to Fig. 1 (left panel), except that the overall median survival was 6.7 months (2 months longer than that observed in the NILCS study). The period elapsed was roughly 15 years (1991–2 to 2007) yielding a median improvement of approximately 4 days a year! Their 1-year survival was $\approx$30% (estimated graphically).

**Fig. 5:**
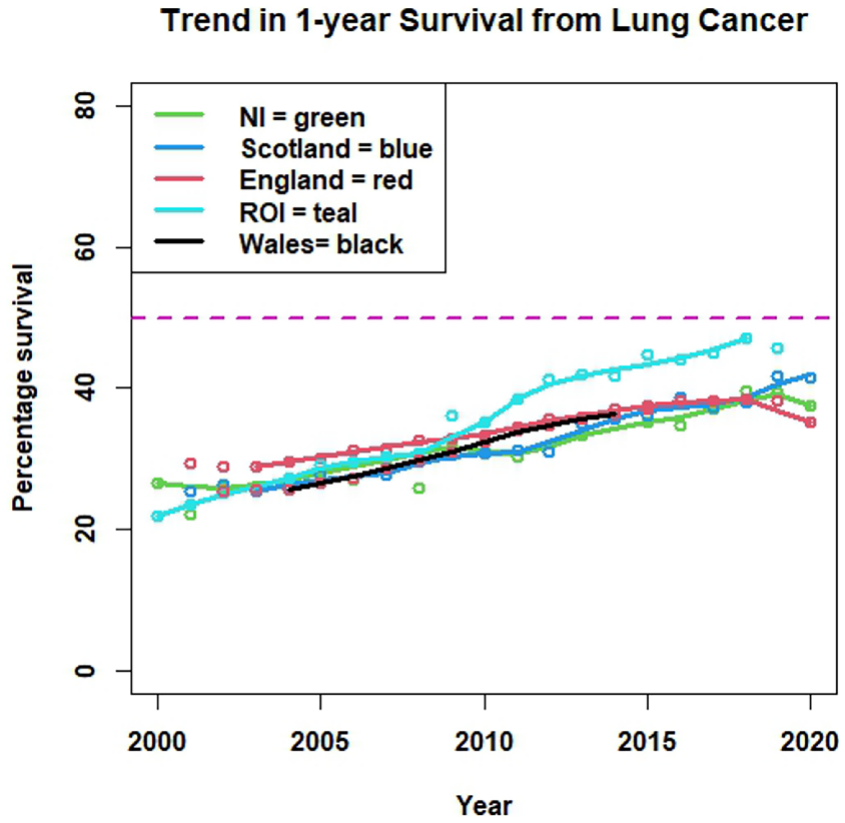
Trends in 1-year survival from lung cancer in regions of the British Isles with smooth regression lines fitted: Welsh trend based on net survival

Figure 5 shows the trends in lung cancer 1-year survival since 2020 in those regions of the British Isles where Cancer Registry data were available [24–28]. The Registries in England, Scotland, NI and the Republic of Ireland provided observed survival, as requested. The Welsh Registry provided relative (i.e.* net*) survival for a shorter period. Their data are based on 5-year rolling averages (e.g. 2002–2006, 2003–2007, etc.), but nevertheless capture the increasing survival trend.^1^ For each region, a smooth regression was fitted to aid interpretation.

There is evidence of increasing survival rates in all regions. The patterns in NI and Scotland are remarkably similar, except in 2020 when the NI rate dips. In England, while improvement was better in earlier years, survival rates peaked in 2018 and declined thereafter (sharply in 2020—by 3%). Wales ranks lower than England (allbeit on a different basis) while survival in the Republic of Ireland is best, reaching a figure of $\approx$ 46% (2018–19): and this from a low baseline in 2000. By contrast, the latest figure for NI (2020) is 37.5%. A useful summary of lung cancer in NI may be found in the Registry’s current fact sheet [29].

## Discussion

The NILCS data are some 30 years old, but the issues raised are evergreen. Unfortunately, the main take home message is that 1-year survival from primary lung cancer remains poor today. This is a disappointing finding given the many improvements in diagnosis, treatment and peri-operative management of lung cancer patients [30–32] since the original study was conducted. It seems that their impact on survival has been less than one might have anticipated, and consequently, earlier detection would seem to offer the best approach to improving survival at this time.

### Methods

Our study was the first, ever, population-based, multi-source referral study of survival amongst incident cases of lung cancer in NI. It is unique in that details were recorded, cross-checked and linked a by a single medical doctor [4]. It is novel because it explicitly acknowledges the non-proportional hazards nature of the survival data encountered by employing the MPR Weibull model, which also models the shape. Burke and MacKenzie [13] have shown that a proportional hazards model produces, in these data, erroneous and misleading confidence intervals for the hazard ratios in the treatment categories by failing to account properly for the waning effect of treatment with time. We also fitted a treatment model to identify the factors used by clinicians to select patients for therapy and aid interpretation of the survival model. Amongst the literature reviewed, only [21] considered a treatment model. In other ways, however, our study is ‘of its time’, and the absence of internationally accepted staging rubrics and other clinical measurements (e.g. grade and tumour size) reflect the absence of a mature cancer registration culture in NI in 1991–2 which, consequently, hampers the interpretation of our results.

### Survival

The NILCS findings clearly indicate that survival from lung cancer remains poor, and only a quarter of patients survived the first year. One-year survival was best in patients receiving surgery, but these were a small, but highly selected, sub-group of all patients. When case-mix factors were allowed for, about half of the benefit due to surgery was attributable to case-mix factors. In any case, in 1991–2, overall survival for lung cancer patients in NI was comparable to that reported elsewhere in the UK.

Fergusson et al. [33], studying a hospital population in South West Scotland, reported a 1-year survival rate of 30%, while [34] had a 12% 2-year survival in a similar population. Findings from the Eurocare Study (1983–5), for England, indicated that the annual average survival rate was 20% [35]—a figure which is lower than that found in NI. During this earlier period, the results for UK were markedly lower than those reported in some parts of Europe, e.g. Finland, France and Holland had 1-year survival figures in the range 39–40%. In Italy, the 1983–5 rate was 30%, and more recent data from a population-based study [36] suggested that the 1-year survival rate, 29%, had hardly changed. Overall, the evidence supports the view that, at that time, 1-year survival rates in NI and the UK lay near the bottom of the European league table.

Since then, survival has improved. When combining all stages of lung cancer in England, for example 1-year survival improved from 24.5% in 1995–9 to 41.6% in 2016 [26, 37]. Much of this improvement has occurred since 2010 and may be attributed to developments in lung cancer care [30]. Figure 5 records improvement in the British Isles: 1-year survival has not quite doubled since the NI study was conducted. Although these increasing trends are welcome, they are rather modest given: (a) the time which has elapsed since the original study and (b) the progress made elsewhere. On the latter point, for cases diagnosed between 2010 and 2014, data on *5-year survival* show the UK (13%) in the bottom third of the European league (cf., Ireland,18%) and bottom of the 5 non-EU countries studied [38]. Thus, it seems that the UK’s relative position is unchanged.

### Treatment models

The use of *risk scores* or *prognostic indices* has been employed by clinicians for some time and may be viewed as *personalizing* medicine. However, some authors tend to restrict this term to genetic profiling in relation to treatment. For example, the recently published 10-year Strategic Plan for Cancer in NI [31] does not mention the use of *statistical modelling* as an adjunct to clinical decision-making in relation to survival and treatment.

Our findings show the usefulness of a treatment model for quantifying the clinical decisions made by doctors in the field by identifying those factors driving their selection of patients for treatment. Inevitably, there is variation between the choices clinicians make (perhaps, *inter alia*, in relation to elderly patients). The screening table quantified considerable discordance in decision-making (both-ways) between doctors and the model. The model also identified larger patient subgroups with improved predicted survival. Patients in the top quartile—with a prob of $\ge 0.75$ of receiving treatment—included almost all of the 79 patients selected for surgery by the clinicians and suggested c120 more for treatment. At that time, there was no opportunity to validate and deploy the model on new data, and thus, its predictions remain untested. However, it is clear that such models, based on current data, may prove useful aids to consistent clinical decision-making in future studies aiming to improve survival.

## Data Availability

The datasets generated during and/or analysed during the current study are available from the corresponding author on reasonable request.

## Footnotes

1 The Welsh Registry expects to make, year-on-year, observed survival trends available later this year.
